# Reconstructing cell-cell interaction network in single-cell spatial transcriptomics via directed heterogeneous graph autoencoder

**DOI:** 10.1093/bioinformatics/btag130

**Published:** 2026-04-17

**Authors:** Jin-Xian Hu, Xiaoyong Pan, Ye Yuan, Hong-Bin Shen

**Affiliations:** Department of Radiology, Ruijin Hospital, Shanghai Jiao Tong University School of Medicine, Shanghai, 200025, China; Institute for Medical Imaging Technology (IMIT), Ruijin Hospital, Shanghai Jiao Tong University School of Medicine, Shanghai, 201801, China; Institute of Image Processing and Pattern Recognition, Shanghai Jiao Tong University, and Key Laboratory of System Control and Information Processing, Ministry of Education of China, Shanghai, 200240, China; Institute of Image Processing and Pattern Recognition, Shanghai Jiao Tong University, and Key Laboratory of System Control and Information Processing, Ministry of Education of China, Shanghai, 200240, China; State Key Laboratory of Biopharmaceutical Preparation and Delivery, Institute of Process Engineering, Chinese Academy of Sciences, Beijing, 100190, China; Institute of Image Processing and Pattern Recognition, Shanghai Jiao Tong University, and Key Laboratory of System Control and Information Processing, Ministry of Education of China, Shanghai, 200240, China

## Abstract

**Motivation:**

Spatial transcriptome data have both gene expression information and cell spatial location information, offering exceptional prospects for analyzing cell-cell interaction (CCI) network. Most existing statistical and optimal transport-based methods rely only on known ligand-receptor pairs to infer CCI network. Furthermore, most current deep learning frameworks rely on symmetric decoders or undirected graph architectures.

**Results:**

Taking advantage of spatial transcriptomic data and graph autoencoders, we present a directed heterogeneous graph autoencoder-based approach DualCellChat to reconstruct a complete and accurate CCI network from incomplete single cell spatial transcriptomics. Benchmarked on five single-cell spatial datasets from four different technologies, we demonstrate that DualCellChat outperforms existing deep learning-based methods and can inherently model the direction of cellular interactions. Furthermore, we introduce downstream analysis to infer signature genes involved in cellular interactions from the reconstructed CCI network and infer significant ligand-receptor pairs for specific cell types.

**Availability and implementation:**

The dataset and code are available in GitHub (https://github.com/JinxianHu/DualCellChat) and Zenodo (DOI: 10.5281/zenodo.18512678).

## 1. Introduction

In multicellular organisms, cells interact with other cells at complex temporal and spatial scales to realize a variety of complex structures and functions. The cells are communicated through specific signaling molecules, including ligands, receptors, structural proteins, and secreted proteins, leading to dynamic changes in cellular functions and inducing structural and functional downstream responses.

The rapid development of spatial transcriptomics technologies has enabled researchers to capture comprehensive gene expression at the single-cell level, while preserving important spatial location information, making it possible to analyze cell-cell interactions at spatial resolution. Currently, there are two main categories of high-throughput spatial transcriptomic technologies that have made important breakthroughs, namely, fluorescence in situ hybridization (FISH) based technologies and RNA sequencing (RNA-Seq) based technologies. FISH-based technologies achieve single-cell resolution with spatial position recording and high-throughput gene expression quantification by combining them with microscopic imaging, such as MERFISH (Moffitt et al. 2018; Xia et al. 2019; Zhang et al. 2021), seqFISH (Eng et al. 2017; Zhu et al. 2018), and seqFISH+ (Eng et al. 2019). RNA-Seq-based technologies obtain molecular information about the whole transcriptome at the individual cellular or subcellular level by using bead, nanoball, or spot to localize spatial locations and sequencing each location, including HDST (Vickovic et al. 2019), Slide-seq (Rodriques et al. 2019), Slide-seqV2 (Stickels et al. 2021), STARmap (Wang et al. 2018), Stereo-seq (Chen et al. 2022), etc.

Several computational tools have been developed for analyzing CCI network with spatial data. Early approaches, such as CellPhoneDB v3 (Garcia-Alonso et al. 2021) and Giotto (Dries et al. 2021), primarily rely on statistical frameworks to identify significant ligand-receptor (L-R) interactions by aggregating co-expression levels within spatial microenvironments. To incorporate physical rigor, transport-based models like SpaOTsc and COMMOT were introduced, modeling intercellular interactions as optimal transport problems (Cang and Nie 2020; Cang et al. 2023). However, most of these methods are constrained by a dependence on known ligand-receptor pairs.

Recently, deep learning has been widely applied to the analysis of spatial transcriptomics, ranging from graph convolutional networks (scstGCN) (Xue et al. 2024) and diffusion models (SpaDiT) (Li et al. 2024) to masked autoencoders (SpaMask, SpaDAMA) (Huang et al. 2025; Min et al. 2025) and multi-view learning (mclSTexp) (Min et al. 2024). By leveraging these deep-learning-based frameworks, several deep graph-based approaches can now reconstruct cell-cell communication directly from transcriptomic data. This shifts the focus toward a discovery of unannotated and non-canonical interactions within high-dimensional space. NCEM (Fischer et al. 2023) uses a graph neural network to relate cell type and spatial information to gene expression. DeepLinc (Li and Yang 2022) employs a variational graph autoencoder to infer missing interactions from incomplete networks. CLARIFY (Bafna et al. 2023) links CCI network with gene regulatory networks, while TENET (Lee et al. 2024) employs a triple-enhancement strategy to denoise sparse spatial networks. More recently, STAGUE (Nie et al. 2024) introduced graph structure learning targeting optimal spatial clustering by refining the spatial adjacency matrix, and VGAE-CCI (Zhang et al. 2025) extended interaction construction to 3D spatial contexts. Despite these advancements, most current deep learning frameworks for CCI reconstruction rely on symmetric decoders or undirected graph architectures, which may fail to capture the asymmetric and directional nature of biological signaling.

To bridge these gaps, we propose DualCellChat, a directed graph auto-encoder that endogenously models CCI directionality to reconstruct a complete interaction network. Specifically, we represent the data as a graph based on spatial neighborhood relationship between cells, where a node represents a cell with the gene expression profile as node attributes, and an edge represents an interaction between cells. To address the direction of cellular transmission signals, DualCellChat learns two feature spaces for cell sending and receiving signals separately in the encoding phase to clarify the dual role of a cell as a source and a target. By parameterizing the graph convolution layer, the source and target nodes are trained to have different importance. In the decoding phase, the learned latent representations by two encoders are combined with the asymmetric inner product to reconstruct the cell-cell interaction network with directions. Finally, considering that there are differences in the expression for different types of cells and differences in the communication between different types of cells, DualCellChat further explores the prior knowledge of cell types to construct the cell-cell interaction network into a heterogeneous graph and learn latent representations of cell interactions based on directed heterogeneous graph convolution.

The DualCellChat model was benchmarked on five single-cell spatial transcriptome data from four different technologies. The experimental results indicate that DualCellChat achieves higher reconstruction accuracy than previous methods DeepLinc, CLARIFY and VGAE-CCI, exhibits high robustness under various data noises. In addition, the incorporation of additional priori knowledge on cell types with the heterogeneous graph convolution is beneficial to further improve the reconstruction accuracy. Furthermore, cross-database validations (including CITEdb, CCIDB, and CCCdb) confirm that DualCellChat consistently identifies biologically enriched CCI network with higher validation rates. DualCellChat also explores the effect of different types of ligand-receptor pairs as inputs for reconstructing the cellular interaction network, showing the deficiencies of using only ligand-receptor pairs to infer cellular interactions. Furthermore, DualCellChat was used to infer ligand-receptor pairs between cell types and potential signature genes that participate in cell interactions. The literature for ligand-receptor pairs shows that the inferred significant ligand-receptor pairs indeed mediate interactions between the two types of cells. And an enrichment analysis of top-ranked signature genes presents a clear enrichment for biological processes associated with cell-cell junction and signaling pathways for short-range cell interactions, separately. The above results suggest that DualCellChat can offer informative clues to better understand cell-cell interactions as well as the spatial organization and functional characteristics of tissues and organs.

## 2. Materials and methods

### 2.1. Datasets

This study utilizes five single-cell spatial transcriptome data (detailed in Table S1 and Supplementary Notes 1), four of which were also used in DeepLinc (Moffitt et al. 2018; Zhu et al. 2018; Vickovic et al. 2019). These four datasets consist of mouse visual cortex data from the seqFISH (Zhu et al. 2018) technology, mouse hypothalamic preoptic region from the MERFISH (Moffitt et al. 2018) technology, and mouse olfactory bulb data and human breast cancer data from the HDST (Vickovic et al. 2019) technology. The spatial transcriptomic techniques used to generate these datasets resulted in varying levels of sparsity in the gene expression profiles. Specifically, the seqFISH and MERFISH platforms cover less than 200 genes, while the HDST platform produces high-throughput transcriptome profiles covering ∼10 000 genes. Nonetheless, the latter two datasets are sparse, as many genes were undetected in many cells. Therefore, we conducted three follow-up experiments using mouse olfactory bulb data generated by Stereo-seq (Chen et al. 2022) technology, which involve incorporating cell type information and ligand-receptor information, as well as downstream cell annotation. This dataset has high cell and gene coverage. By encompassing diverse species (mouse and human) and tissue types (different normal brain regions and cancerous tissues), the benchmark datasets used in this study represent a wide range of biological contexts.

The cell type annotations adopted in this study for SeqFISH, MERFISH, and HDST datasets were derived from their original papers, similar to DeepLinc, to facilitate convenient comparison. All of these studies combined spatial transcriptomics technologies with scRNA-Seq data to annotate cell types. For the Stereo-seq dataset, cell type annotations were performed using toolkit IRIS (Ma and Zhou 2024) and STAGATE (Dong and Zhang 2022), the visualization of layered structure and annotations can be found in Fig. S1.

### 2.2. Graph construction and data preprocessing

In solid tissues, adjacent cells in direct spatial contact are more likely to have certain types of interactions than randomly selected, spatially distant non-adjacent cells (Li and Yang 2022). The identification of adjacent contact cell pairs is based solely on the geometric proximity of individual cells in the spatial space.

Consequently, a cell-cell interaction network in direct contact can be represented as a cell adjacency network G (A, X), in which nodes represent cells and edges indicate direct contact between cells. The adjacency matrix is denoted by the matrix A, and the nodes are characterized by the gene expression of a single cell, which is represented by the matrix X ∈ R^N × M^ (with N cells and M genes). The cell-neighborhood matrix A is derived from the spatial geometric distances between cells within a tissue section. To determine which pairs of cells are in direct contact within the tissue section, we calculate the distance between each pair of cells and the distance between each cell and its three nearest neighbors. Based on the two distributions described above, distance thresholds were established for pairs of cells in direct contact within each tissue section (Li and Yang 2022). For each cell, only the three nearest neighbors within the distance threshold were defined as direct contact interactions. Then, all cell pairs that satisfy the condition form the neighborhood matrix A. The cell adjacency network G, constructed using the cell-neighborhood adjacency matrix, represents a positive subset of the cell-cell communication network. This work focuses on learning from this incomplete network and reconstructing a comprehensive cell-cell interaction network.

### 2.3. The DualCellChat framework

**Figure 1:**
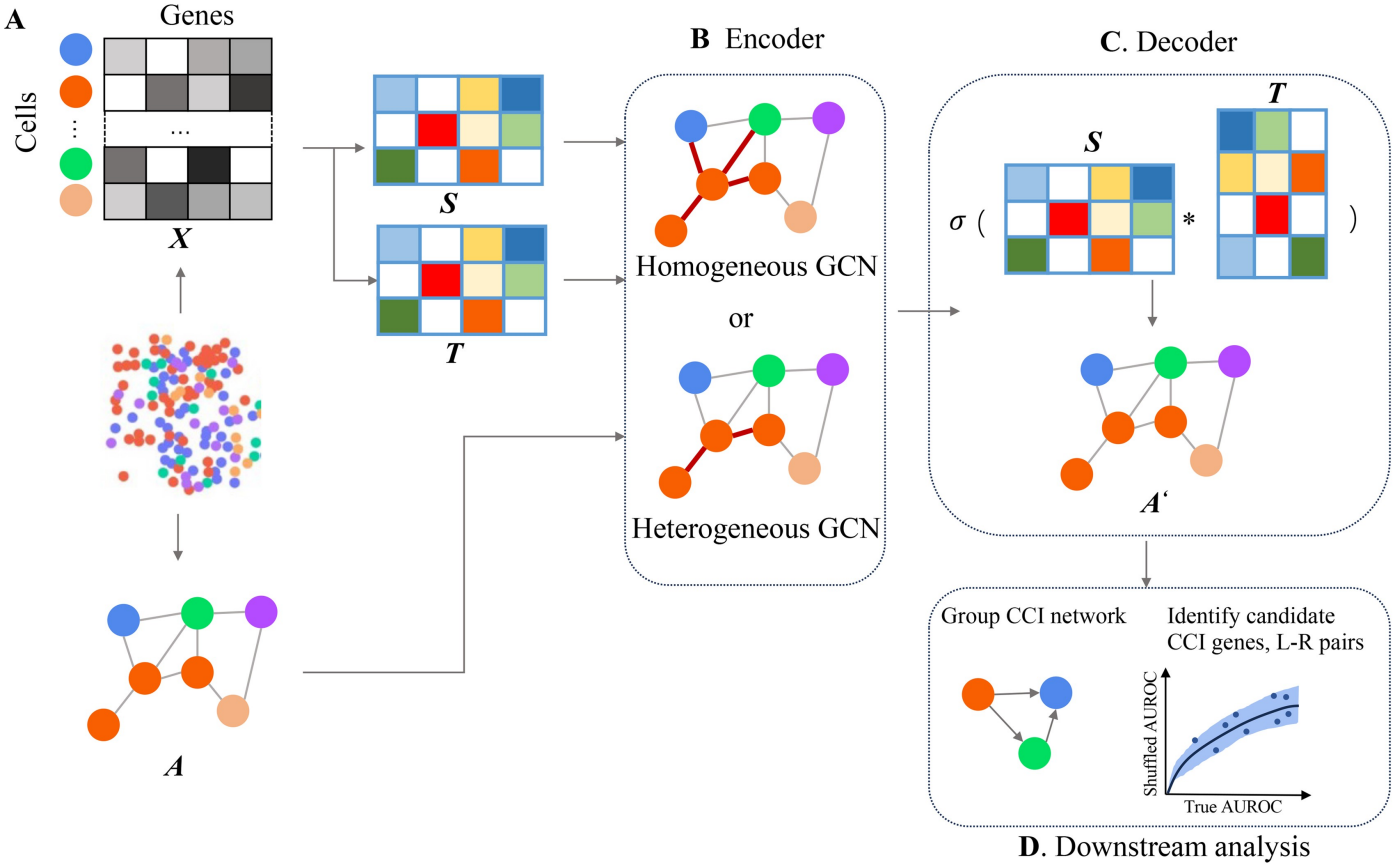
Framework of DualCellChat. (A) data preprocessing. Cellular adjacency graph is represented by G = (X, A), where X and A denote the feature matrix and the adjacency matrix, respectively. X is generated from the gene expression information and A is generated based on the spatial location of the cells. S and T denote the sending and receiving signal characterization to be learned, respectively, to clarify the dual role of a cell as a source and target. Before model training, both S and T are initialized by the feature matrix X. (B) Encoder, specifically the directed graph convolution network, can be trained to update S and T, using either homogeneous graph convolution layers (without cell type information) or heterogeneous graph convolution layers (with cell type information). (C) Decoder, utilizing asymmetric inner product, reconstructs the cell-cell interaction network A′ based on the updated S and T from the encoder. (D) Downstream analysis, including Group CCI network visualization according to cell type, as well as signature genes and ligand-receptor pairs identification based on reconstructed cell-cell interaction graph A′.

In this study, we proposed DualCellChat to reconstruct the cell-cell interaction network using spatial single cell transcriptome data. As shown in Fig. 1, the genes expression and the spatial location of cells are simultaneously used as inputs to construct a graph, DualCellChat simultaneously learns the cell communication mechanism from the gene expression matrix and cellular adjacency matrix through directed graph autoencoder. DualCellChat employs a series of directed graph convolution layers (homogeneous or heterogeneous graph convolution network) to learn intercellular communication mechanisms from the graph (cell location), including node attributes (gene expression), and edges (intercellular interaction) in the encoding phase; and reconstructs a complete and accurate cellular interaction network (reconstructed edges) in the decoding phase.

#### 2.3.1. Graph convolution and reconstruction

**Directed graph convolutional layer for homogeneous graphs.** The encoder in DualCellChat utilizes a spectral-based directed graph convolution network (GCN) from DiGAE (Kollias et al. 2022). The mathematical definition of directed graphs is provided in Supplementary Notes 2. This directed GCN employs a variant of the Weisfeiler-Leman algorithm that emphasizes the dual role of the graph nodes as both senders and receivers. To capture these asymmetric roles, the model represents each node i with a pair of embeddings: a sender embedding $s_{i}$ and a receiver embedding $t_{i}$, which characterize the node’s functional capacity to send and receive signals, respectively. These embeddings constitute the i-th rows of the sender matrix S and receiver matrix T. Consistent with the DiGAE framework, both S and T are initialized from the input gene expression matrix X (i.e. $S^{0}=T^{0}=X$).

During the training phase, S and T evolve through an alternating propagation mechanism. Specifically, to update the sender embedding of node i, the model aggregates messages from the receiver embeddings of its out-neighbors $N^{+}(i)$ (nodes it points to). Conversely, to update its receiver embedding, it aggregates messages from the sender embeddings of its in-neighbors $N^{-}(i)$ (nodes pointing to it). This mechanism mirrors the bipartite message passing in the 1-WL extension, where information flows across distinct source and target channels. To implement this mechanism within a GCN framework, the propagation is controlled by two independent learnable weight matrices, $W_{S}$ and $W_{T}$. Mathematically, the t-th directed graph convolutional layer updates the sender embedding S by aggregating the embeddings of the neighboring receiver nodes T as:


(1) $$S^{\left(t+1\right)}\leftarrow {{(\tilde{D}}^{+})}^{-\beta }\tilde{A}{{(\tilde{D}}^{-})}^{-\alpha }T^{\left(t\right)}{W_{T}}^{\left(t\right)}$$

where ${\tilde{D}}^{+}$ and ${\tilde{D}}^{-}$ denote the degree matrices corresponding to out-degrees and in-degrees on the diagonal of a directed graph, and ${W_{T}}^{\left(t\right)}$ are the learnable parameters for the receiver nodes. The parameters α and β are tunable parameters used to weight the degrees in message passing. Similarly, the receiver nodes embeddings T are updated by aggregating the sender nodes embedding S in the neighborhood:


(2) $$T^{\left(t+1\right)}\leftarrow {\left({\tilde{D}}^{-}\right)}^{-\alpha }{\tilde{A}}^{⊤}{{(\tilde{D}}^{+})}^{-\beta }S^{\left(t\right)}{W_{S}}^{\left(t\right)}$$

where ${W_{S}}^{\left(t\right)}$ are the learnable parameters for the sender nodes.

If we define $\hat{A}={{(\tilde{D}}^{+})}^{-\beta }\tilde{A}{{(\tilde{D}}^{-})}^{-\alpha }$, the following transformation formulas can be used to compactly represent the directed graph convolutional layer:


(3) $$S^{\left(t+1\right)}\leftarrow {\hat{A}T}^{\left(t\right)}{W_{T}}^{\left(t\right)}$$

(4) $$T^{\left(t+1\right)}\leftarrow {\hat{A}}^{⊤}S^{\left(t\right)}{W_{S}}^{\left(t\right)}$$

**Directed convolutional layer for heterogeneous graphs.** To model complex biological systems, DualCellChat extends its framework to a heterogeneous graph by incorporating prior cell type information. Since a tissue typically contains multiple types of cells, and different types of cells interact with each other differently. Constructing a cellular interaction network as a heterogeneous graph network can account for the heterogeneity among cells.

Unlike homogeneous graph that apply a single set of $W_{S}$ and $W_{T}$ globally, the heterogeneous directed convolution accounts for the distinct semantic spaces of different cell populations. To implement this heterogeneity, the propagation is controlled by a set of type-specific learnable weight matrices, ${W_{S}}^{k}$ and ${W_{T}}^{k}$. By integrating the node-type parameter matrix (Wang et al. 2019) into the spectral-based directed convolution framework, this approach enables a message-passing mechanism that captures the unique signaling patterns inherent to specific cell-cell pairs. Mathematically, the t-th heterogeneous directed layer updates embeddings by projecting them into type-aware latent spaces, preserving the functional heterogeneity of the tissue architecture:


(5) $$S^{\left(t+1\right)}\leftarrow {\hat{A}T}^{\left(k,t\right)}{W_{T}}^{\left(k,t\right)}$$

(6) $$T^{\left(t+1\right)}\leftarrow {{\hat{A}}^{⊤}S}^{\left(k,t\right)}{W_{S}}^{\left(k,t\right)}$$

where $T^{\left(k,t\right)}$ and $S^{\left(k,t\right)}$ are the embedding of nodes according to their cell type k, and ${W_{T}}^{\left(k,t\right)}$ and ${W_{S}}^{\left(k,t\right)}$are the learnable parameter matrices for the node type.

**DualCellChat model.** DualCellChat defines an encoder by stacking two nonlinear layers, which connect directed graph convolutional layers. The decoder is to compute the inner product of the source coding matrix S and the target coding matrix T. For example, the encoder using a homogeneous graph can be represented as:


(7) $$Z_{S}=\hat{A}\mathrm{ReLU}({\hat{A}}^{⊤}S^{\left(0\right)}W_{S}^{\left(0\right)}){W_{T}}^{\left(1\right)}$$

(8) $$Z_{T}={\hat{A}}^{⊤}\mathrm{ReLU}(\hat{A}T^{\left(0\right)}W_{T}^{\left(0\right)}){W_{S}}^{\left(1\right)}$$

After the training of the encoder, DualCellChat employs an asymmetric inner product decoder to infer missing directed links from the limited and incomplete cell-cell interaction network. And the decoder used to compute the reconstructed adjacency matrix $A^{\prime }$ is:


(9) $$A^{\prime }=\sigma (Z_{S}{Z_{T}}^{⊤})$$

#### 2.3.2. Reconstructing the cellular adjacency graph

As previously described, DualCellChat inferred the probability of interactions among all cell pairs. Next, cell pairs exhibiting high probability scores were formed the reconstructed cell-cell interaction network. In the following, the accuracy and robustness of the DualCellChat model for reconstructing the cellular adjacency matrix were verified in several cases.

1. **Comparison with existing deep learning algorithms.** The performance of DualCellChat was evaluated against DeepLinc (Li and Yang 2022), CLARIFY (Bafna et al. 2023), and VGAE-CCI (Zhang et al. 2025) in reconstructing the cell adjacency network. To ensure a fair comparison, all methods were trained and tested on the same datasets using identical splits. The edges within the cell adjacency network were randomly partitioned into two subsets: 90% for training and 10% for testing. Negative samples for testing comprised pairs of randomly chosen cells that were not adjacent on tissue slices.
2. **The effect of adding artificial noise on the reconstruction of cellular interactions.** Random noise was independently generated and applied to each gene in every single cell, with new gene expression created by adding the noise as follows:
  (10) $$\frac{{\mathrm{Exp}}_{\mathrm{noise}+}(i,j)}{{\mathrm{Exp}}_{\mathrm{normal}}(i,j)}=2^{\mathrm{rand}}$$
   
  (11) rand∼N(0,σ)
  where ${\mathrm{Exp}}_{\mathrm{noise}+(i,j)}$ indicates the simulated expression value of gene j in cell i after adding noise, and ${\mathrm{Exp}}_{\mathrm{normal}}(i,j)$ indicates the original expression of gene j. To simulate noise, we used the fold change same with DeepLinc (Li and Yang 2022), which follows a normal distribution N(0,σ) after $\log_{2}$ transformation. Different σ values result in varying levels of random noise.
3. **The Effect of missing edges on the reconstruction of cellular interactions.** In this task, edges from the original cell adjacency network were randomly removed at varying percentages. The remaining edges served as the training data for DualCellChat. In this experiment, an equal number of originally non-existent edges were randomly generated as a collection of true negative samples.
4. **The Effect of gene expression dropout on the reconstruction of cellular interactions.** Single-cell spatial transcriptome data is frequently influenced by high sparsity caused by gene expression dropout. To investigate the impact of gene expression dropout, we modeled two scenarios. In the first scenario, varying proportions of genes were randomly eliminated from gene expression profiles to simulate gene dropout. In the second scenario, different proportions of non-zero gene values were randomly selected and set to zero to simulate the dropout of individual genes in a single cell.
5. **The Effect of using only ligand-receptor genes on the reconstruction of cellular interactions.** In this work, the ligand-receptor pair information provided in CellChatDB is utilized as an a priori knowledge to be added to the training of the DualCellChat model to explore the effect of using only ligand-receptor genes versus all genes as inputs on the reconstruction of cell-cell interactions. When only ligand-receptor data is used as input, the ligand gene expression and receptor gene expression matrices are used to initialize the S and T matrices in the DualCellChat model, respectively.

#### 2.3.3. Assessment of interactions between specific cell types

A previously established strategy was utilized to assess the enrichment of interactions between various cell types or within a single cell type (Dries et al. 2021; Li and Yang 2022). Specifically, reconstructed single-cell intercellular interaction networks were randomly shuffled 1000 times to construct a null distribution. In these 1000 shuffled networks, the number of cell interactions of a certain type, or interactions between two cell types served as the null distribution. The number of observed interactions between the cell types to be focused is then compared to this null distribution, and a *P*-value is calculated. This *P*-value represents the frequency with which the randomly generated value would exceed the observed value, thereby reflecting the enrichment of the network for a particular type of interaction.

#### 2.3.4. Screening for signature genes participated in intercellular interactions

Sensitivity scores (Li and Yang 2022) were used to assess the potential impact of a gene on the reconstruction of the intercellular interaction network. For each specific gene in the single-cell spatial transcriptome data, its expression in all single cells was randomly shuffled. The expression of one gene was shuffled at a time, and this newly generated expression data with one specific shuffled gene was then fed into DualCellChat to reconstruct a new cellular interaction network. The reconstructed network generated from the two inputs, the original gene expression profile and the shuffled expression profile of one gene, was then compared, and the difference (ΔAUROC) between the reconstructed AUROC values of the two inputs was computed as the significance score of the gene for the reconstructed intercellular interaction network. This process was repeated 30 times for each gene in the spatial transcriptome dataset, and the average value of ΔAUROC was used as the final sensitivity score to quantify the reconstructed network’s sensitivity to that gene.

The potential signature genes inferred to participate in cellular interactions, as identified by DualCellChat, were subsequently validated through enrichment analysis. Sensitivity scores were calculated for all genes in the two high gene throughput HDST datasets. The top-ranked signature genes were then subjected to Gene Ontology (GO) enrichment analysis to identify the biological processes. To perform functional enrichment analysis, an online analysis website (Gene Ontology Resource, http://geneontology.org/) was utilized in this study.

## 3. Results

### 3.1. Applying DualCellChat to single-cell spatial transcriptome data

According to a previous study (Li and Yang 2022), if two cells are sufficiently close in spatial solid tissues, then there is a direct-contact interaction between these two cells. This direct-contact cellular interaction defined by spatial geometric proximity is only a portion of the complete cell-cell interaction network, containing missing interactions. In addition, gene expression in cells is often compromised by significant noise, such as artificial noise and dropout of gene expression. Therefore, DualCellChat needs to learn from both cellular spatial information and noisy single-cell transcriptome information, and reconstruct a complete and accurate cell-cell interaction network. DualCellChat was applied to four datasets to evaluate the performance of reconstructing cell adjacency network, namely the seqFISH, MERFISH, HDST mouse olfactory bulb, and HDST breast cancer datasets.

**Figure 2:**
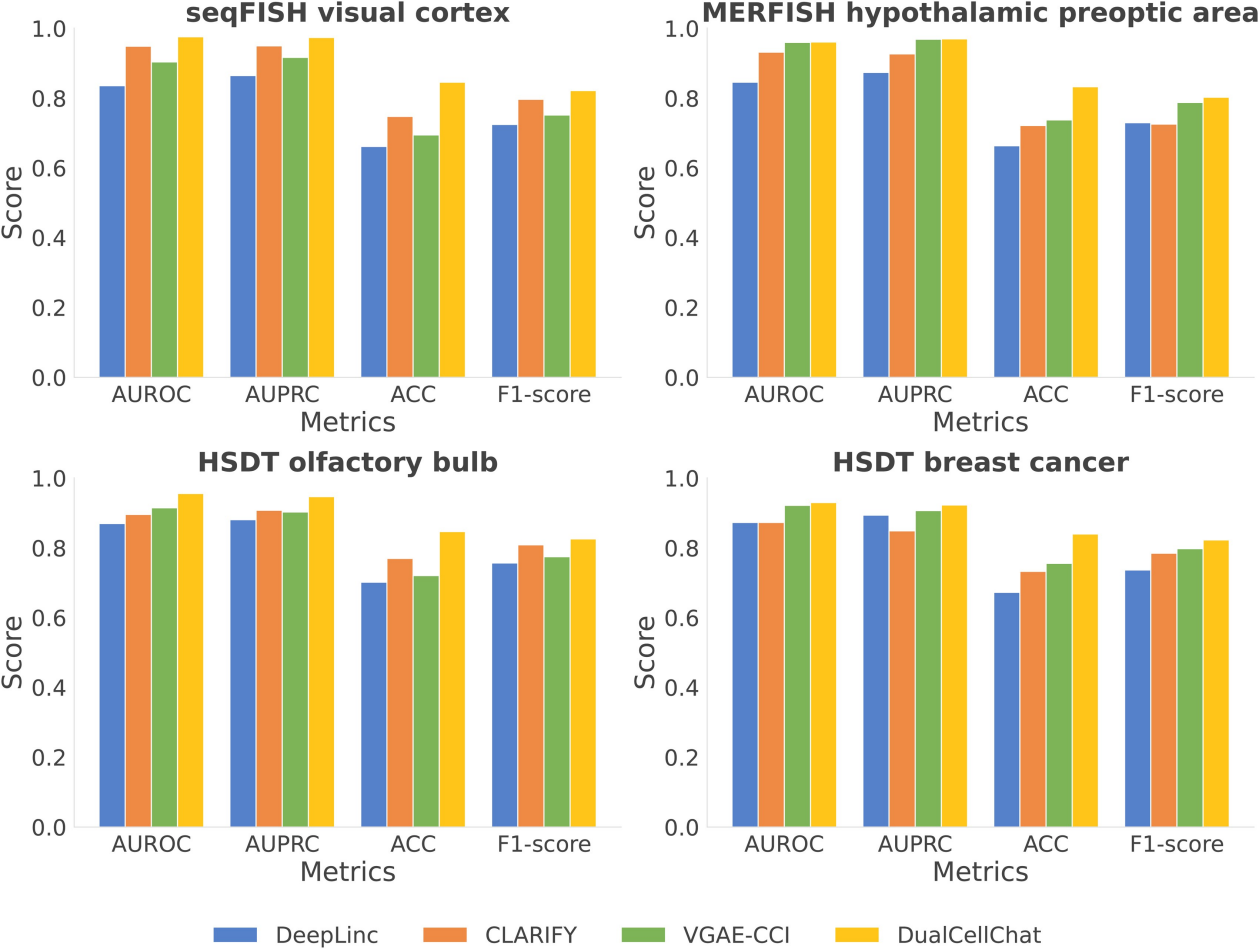
Benchmarking of reconstructing cellular adjacency graph. Comparison of DualCellChat with DeepLinc, CLARIFY, and VGAE-CCI across four spatial transcriptomics datasets.

First, DualCellChat’s reconstruction performance for edges in direct contact was compared with three state-of-the-art methods: DeepLinc (Li and Yang 2022), CLARIFY (Bafna et al. 2023), and VGAE-CCI (Zhang et al. 2025) (Fig. 2). The performance was comprehensively assessed across four metrics: Area Under the Receiver Operating Characteristic (AUROC), Area Under the Precision-Recall Curve (AUPRC), F1-score, and Accuracy. Across all four single-cell datasets, DualCellChat consistently demonstrated superior performance.

**Figure 3:**
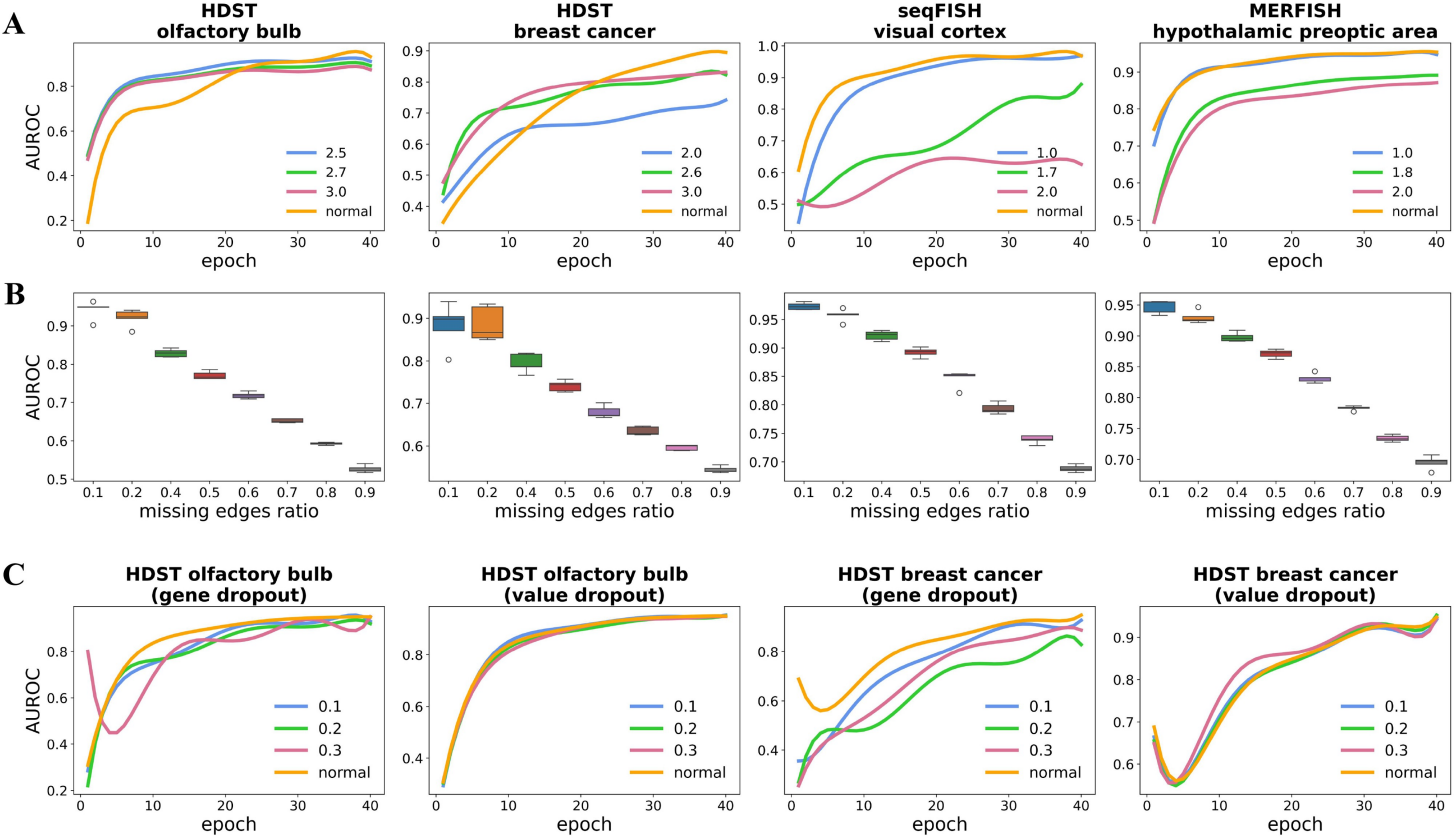
Accuracy of reconstructing cellular adjacency graph. (A) Effect of artificial random noise on DualCellChat reconstruction results. (B) Effect of random removal of real edges on DualCellChat reconstruction results. (C) Effect of gene expression dropout on DualCellChat reconstruction results, including gene dropout and gene expression dropout (value dropout) of a single data point in a cell.

Next, experiments were conducted to evaluate DualCellChat’s tolerance to simulated noise in single-cell gene expression data. To be specific, random perturbations with different levels of Gaussian distribution were introduced into the gene expression profiles of all cells (Equations 10 and 11). As shown in Fig. 3A and Fig. S2, the performance of DualCellChat remained stable despite the addition of random noise, demonstrating its stability in the presence of noisy gene expression data. This characteristic is particularly important when analyzing single-cell spatial transcriptomic datasets. Nonetheless, the performance of DualCellChat declines with further corruption of the expression data, implying that DualCellChat requires a certain level of gene expression information to accurately reconstruct cellular interactions. Additionally, to determine the noise level at which DualCellChat’s results would significantly decline, statistical significance testing was employed. Specifically, for each noise level, DualCellChat was run five times, and the reconstruction accuracy was recorded. An independent samples *t*-test was performed between the results at each noise level and the original results. If the *P*-value was less than the significance level (e.g. 0.05), the change in performance was considered significant. Table S2 presents the *P*-values under different noise levels. The results show that when statistical significance was observed (*P*-value < 0.05), the noise levels for HDST_ob and HDST_cancer were relatively higher (2.7 and 3.3, respectively), while those for seqFISH and MERFISH were lower (1.2 and 1.5, respectively).

Furthermore, we evaluated the capacity of DualCellChat to reconstruct intercellular interactions under various ratios of missing edges. To be specific, a certain ratio of current edges was arbitrarily eliminated from the original cell adjacency network. Then, the DualCellChat model was trained on the remaining edges and gene expression data from the single-cell transcriptome. Ultimately, AUROC values were calculated to assess DualCellChat’s ability to accurately identify arbitrarily removed intercellular interaction edges (positive edges) from interaction edges that were not originally present (negative edges). As illustrated in Fig. 3B, DualCellChat achieved a higher accuracy in reconstructing missing positive intercellular interactions. For instance, even if 50% of the interaction edges were eliminated in the original cell adjacency network, DualCellChat could still reconstruct these missing edges with over 70% accuracy. This result demonstrated that DualCellChat could effectively learn from incomplete intercellular interactions to reconstruct missing edges. This capability was essential for DualCellChat to deduce the complete intercellular communication networks from the incomplete spatial cell adjacency network.

Finally, we explored DualCellChat’s tolerance for dropout phenomena in gene expression data. For various technical reasons, single-cell transcriptome data usually suffer from the dropout phenomenon of gene expression, as seen in HDST data. Therefore, we simulated two types of gene expression dropouts in HDST data, namely the dropouts of genes and the dropouts of expression of a single data point in a cell. In the first type of dropouts, different proportions of genes were arbitrarily deleted from the expression matrix; in the second type of dropouts, different proportions of non-zero gene values were arbitrarily selected and set to zero. DualCellChat showed a high tolerance for both types of dropouts in gene expression data (Fig. 3C). As expected, more severe dropouts of gene expression values decreased the accuracy of DualCellChat.

Taken together, the results validate the advantages of DualCellChat in deriving potential information about cell-cell interactions from highly sparse and noisy spatial information. In addition to these accuracy and robustness evaluations, the biological interpretation and downstream cell annotation analysis of the learned S and T representations are provided in Supplementary Notes 4 and 5.

### 3.2. Integrating cell type prior information with heterogeneous graph convolution

Different types of cells exhibit variations in gene expression and cellular function, as well as differences in interaction with other types of cells. To further investigate the impact of incorporating prior cell type information on the reconstruction of intercellular interaction, this work proposes directed heterogeneous graph convolution as an encoder model. Cell type was used as an additional label to construct a heterogeneous graph for cell-cell interaction networks, where cell type represents the type of node. Fig. S3 show the results of reconstructing the cell adjacency network based on directed homogeneous graph convolution (i.e. directed graph convolution used in most of the experiments in this work) and directed heterogeneous graph convolution on Stereo-seq data. Performance was also compared against baselines where cell types were simply used as node features in a homogeneous GCN, as well as models using randomly initialized cell annotations in a heterogeneous setting. The results indicated that the reconstruction accuracy of cellular interactions can be improved by adding accurate cell types as additional information to the model and learning the feature space of different cells separately. Furthermore, quantitative analysis using UMAP-based variance (Table S3) demonstrates that the heterogeneous module is most beneficial for datasets with high intra-type diversity and unbalanced biological complexity. Mean Distance to Centroid measures cell-to-centroid Euclidean distances in UMAP space, using mean and standard deviation to reflect cluster tightness. UMAP Variance quantifies intra-cluster heterogeneity (Mean) and cross-lineage “polarization” (standard deviation) based on spatial dispersion.

### 3.3. Visualizing interactions between cell types

**Figure 4:**
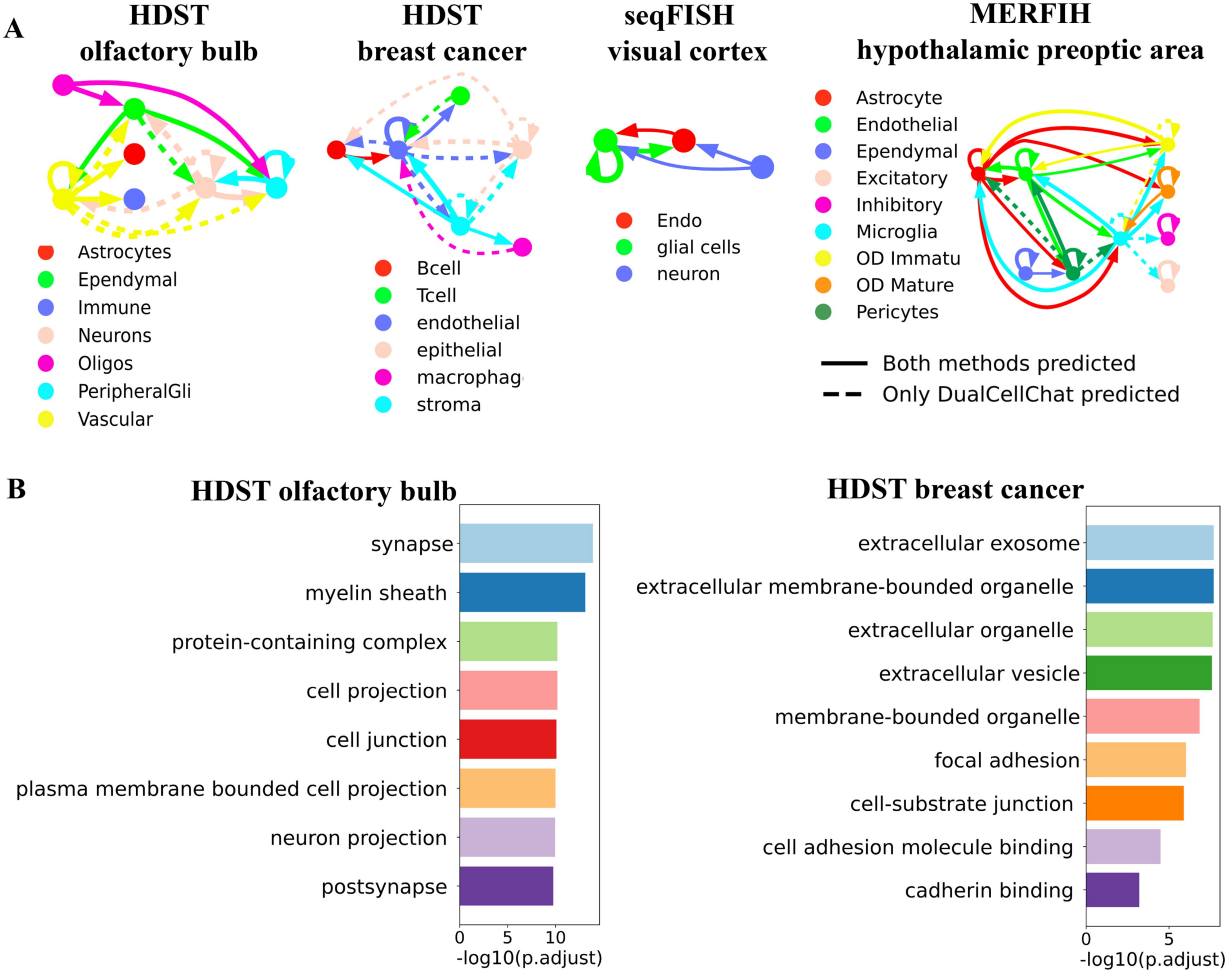
(A) Visualization of enriched cell-cell interactions with directions reconstructed by DualCellChat according to cell type. Four datasets were used to evaluate the enriched interactions between cell types in the reconstructed cell communication networks. Nodes represent cell types, and colors indicate different cell types. The lines with arrows depict the enriched communication between cell types. (B) Biological processes enriched by the signature genes inferred by DualCellChat. Gene Ontology enrichment analysis was performed on the genes with the highest sensitivity scores in the two datasets of HDST.

To evaluate the reconstructed intercellular communication networks, we employed a permutation test to examine the enrichment of interactions between specific cell types in the reconstructed network. We compared the observed enrichment of cell types of interactions to randomly assembled networks with the similar magnitude. Fig. 4A illustrates the enriched intercellular interaction network reconstructed by DualCellChat. In this study, we compared the enriched intercellular interactions reconstructed by DualCellChat and DeepLinc. To validate our predictions, we cross-referenced them against three public cell-cell interaction databases, CITEdb (Shan et al. 2022), CCIDB (Noh et al. 2023), and CCCdb (Xu et al. 2026), showing higher overlap rates for DualCellChat than DeepLinc across multiple tissue contexts. Results showing that DualCellChat consistently achieves higher validation rates than DeepLinc across multiple datasets: 41.8% (7/17) vs. 22.7% (5/22) in HSDT_cancer, 17.7% (3/17) vs. 5.9% (2/34) in HSDT_ob dataset, 100% (5/5) vs. 55.5% (5/9) in seqFISH, and 28.1% (9/32) vs. 17.4% (8/46) in MERFISH. Detailed information is available in the Table S4.

### 3.4. Identifying signature genes participated in short-range cell interactions

We conducted a further evaluation of the DualCellChat to identify signature genes that play significant roles in reconstructing intercellular interaction networks. Specifically, we randomly shuffled the expression of a specific gene in all single cells and then input the modified transcriptome data and cell adjacency network into DualCellChat.

The sensitivity score (Li and Yang 2022) of the DualCellChat model for a specific shuffled gene serves as the contribution of that gene to intercellular interactions. We performed this calculation for individual genes to derive sensitivity scores for all genes. Due to the low gene coverage of the seqFISH and MERFISH datasets, only the two high-throughput datasets from HDST were analyzed in this study. DualCellChat identified signature genes with high sensitivity scores that are associated with cellular processes and molecular functions involved in direct cell-cell interactions (Liu et al. 2022) (Fig. 4B). We performed GO enrichment analysis on the top 200 genes with the highest sensitivity scores. The genes exhibiting the highest sensitivity scores were found to be associated with biological processes and cellular components related to cell-cell and cell-substrate junctions. These biological processes and cellular components are critical for regulating the formation and stability of cell-cell contacts. Subsequently, we searched the literature to further validate the potential signature genes involved in intercellular interactions. More details can be found in the Supplementary Notes 3. Based on the above results, DualCellChat has the capability to identify potential signature genes that are directly or indirectly involved in short-range intercellular interactions. By utilizing the DualCellChat framework, we can gain insights into key molecules that mediate interactions between different cell types.

### 3.5. Results of ligand-receptor pairs used to reconstruct the cellular adjacency graph

In this section, only the ligand-receptor genes are used as inputs to reconstruct the cellular neighboring matrix defined by spatial geometric distances. CellChatDB (Jin et al. 2021) categorizes the ligand-receptor pairs into Cell-Cell Contact, Secreted Signaling, and Extra Cellular Matrix, based on known protein structures and biological pathways. Here we evaluate the results of reconstructing the cell-neighborhood matrix using each of these three types of ligand-receptor pairs as input. Due to the DualCellChat model’s discrimination between the dual roles of cellular sending and receiving signals, it is possible to differentiate the signal space at the outset by using the ligand gene expression to initialize the S-matrix for sending signals, and the receptor gene expression matrix to initialize the T-matrix for receiving signals, respectively.

**Figure 5:**
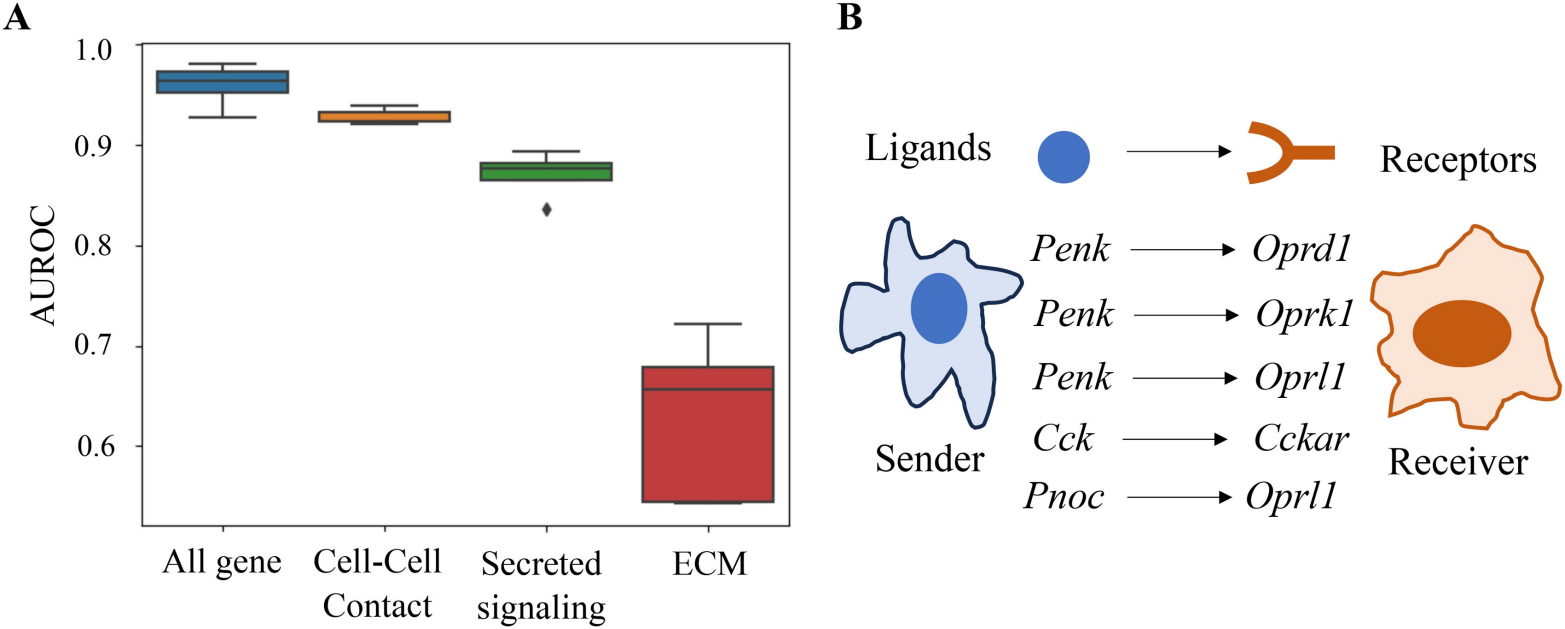
(A) Reconstruction results using only ligand-receptor genes as input and using all genes as input to DualCellChat on Stereo-seq data. The ligand-receptor genes consist of three categories: Cell-Cell Contact, Secreted Signaling, and Extra Cellular Matrix (ECM). Among them, the ligand gene expression was used to initialize the S-matrix for sending signals, and the receptor gene expression matrix was used to initialize the T-matrix for receiving signals. (B) Identifying signature ligand-receptor pair in cell-cell interactions.

Figure 5A demonstrates the comparison of results using each of the three ligand-receptor pairs versus using all genes as inputs in on Stereo-seq dataset. Interestingly, the ligand-receptor pair using cell-cell contacts can achieve the best reconstruction accuracy among the three types of ligand-receptor pairs and is close to the reconstruction results using all genes. This leads to two conclusions: (i), the results using ligand-receptor pairs with cell-cell contacts are the best among the three types of ligand-receptor pairs, which is consistent with what we expected. Interactions constructed from spatial geometric distances focused on spatially proximate cells, and these interactions were themselves preferred to ligand-receptor interactions by cell-cell contacts. (ii), the use of ligand-receptor pairs alone did not achieve the same results as the full range of genes, potentially suggesting that information about cell-cell interactions is not limited to specific known ligand and receptor genes. Thus, we expand the cellular communication defined by ligand-receptor pairs by using the DualCellChat to calculate sensitivity scores for the full range of genes, further identifying additional signature genes potentially involved in cellular communication.

### 3.6. Identifying signature ligand-receptor pair in cell-cell interactions

To further investigate the molecular drivers of cell-cell communication, we identified L-R pairs that contribute significantly to the interactions between specific cell types across seqFISH, MERFISH, HDST_ob and HDST_cancer datasets. For each dataset, L-R pairs were filtered from the CellChatDB (Jin et al. 2021) that were co-expressed in the corresponding cell types. Then, for each ligand-receptor pair, sensitivity score was calculated using a process like the screening of signature genes described in Section 2.3.4. Specifically, for each ligand-receptor pair, the expression of the gene pair in all cells was randomly shuffled at the same time, and then the modified transcriptome data were fed into DualCellChat and the reconstructed results were compared to the original AUROC values obtained from the unmodified data. Calculations were performed for all preserved ligand-receptor pairs, and the ligand-receptor pairs were then sorted according to sensitivity scores. The comprehensive results for four datasets are provided in Table S5. As an example, Fig. 5B illustrates the top five predicted interactions between Microglia and Astrocytes in the MERFISH dataset. To validate these high-ranking ligand-receptor pairs, we performed a literature search. A recent study (Wheeler et al. 2023) confirmed that the astrocyte receptor *Oprl1* transduces signals from the microglia ligand *Pnoc*. Similarly, in the T cell-endothelial cell prediction for the HDST_cancer dataset, the ICAM1-ITGB2 pair ranked seventh. This ICAM1-ITGB2 pair is supported by earlier findings (Marlin and Springer 1987) which demonstrated that LFA-1 (ITGB2) acts as a receptor for ICAM-1, mediating adhesion between T cells and the endothelium.

## 4. Discussions

This study introduces DualCellChat, a deep generative model based on the directed graph autoencoder, which can reconstruct a comprehensive intercellular communication network from single-cell transcriptomic data. DualCellChat considers the dual role of cells as both a source and a target of signals by learning the two feature spaces separately. Due to the asymmetry between the two feature matrices, DualCellChat can reconstruct intercellular interactions with directional information through an asymmetric inner product as a decoder. Additionally, the graph convolution layer is parameterized to assign different importance to the source and target nodes.

Experimental results on multiple real single-cell transcriptome datasets demonstrate that DualCellChat is capable of learning from incomplete intercellular communication networks, reconstructing a comprehensive cell-to-cell interaction network, and achieving a high accuracy, and identifying the direction of cellular communications. Moreover, DualCellChat is memory-efficient and scalable, making it suitable for large single-cell datasets (Supplementary Note 6). The cellular communication representations learned by DualCellChat can also be used to infer signature genes participated in cellular interactions. Furthermore, this study examines the effects of incorporating additional prior information, such as ligand-receptor gene information and cell type information, on the reconstruction of cell adjacency networks. In particular, we utilize cell type information to construct the intercellular interaction network as a heterogeneous graph and propose a novel directed heterogeneous graph convolution model. This model is designed to learn distinct feature spaces for different cell types, thereby enhancing the accuracy of intercellular interaction network reconstruction. In conclusion, as a deep directed heterogeneous graph autoencoder-based method, DualCellChat offers unique advantages in fully leveraging single-cell spatial transcriptomic data with directional information and additional cell type information, identifying potential features, and uncovering potential signature genes involved in cellular interactions.

## Supplementary Material

btag130_Supplementary_Data

## Data Availability

All datasets utilized in this study are publicly available in Zenodo, at https://zenodo.org/records/18512678.
