# Supplementary material for: Reconstructing cell-cell interaction network in single-cell spatial transcriptomics via directed heterogeneous graph autoencoder
Source: Bioinformatics. 2026 Apr 17;42(5):btag130. doi: 10.1093/bioinformatics/btag130 (PMC13189858; doi:10.1093/bioinformatics/btag130)
Supplement: btag130_Supplementary_Data [file btag130_supplementary_data.zip › supplementary_materials/supplementary materials.docx]

**Supplementary Materials**

For article:

**Reconstructing cell-cell interaction network in single-cell spatial transcriptomics via directed heterogeneous graph autoencoder**

Jin-Xian Hu^1, 2, 3^, Xiaoyong Pan^3^, Ye Yuan^4,*^, and Hong-Bin Shen ^3,*^

^1^Department of Radiology, Ruijin Hospital, Shanghai Jiao Tong University School of Medicine, Shanghai, 200025, China

^2^Institute for Medical Imaging Technology (IMIT)，Ruijin Hospital, Shanghai Jiao Tong University School of Medicine, Shanghai, China

^3^Institute of Image Processing and Pattern Recognition, Shanghai Jiao Tong University, and Key Laboratory of System Control and Information Processing, Ministry of Education of China, Shanghai 200240, China

^4^State Key Laboratory of Biopharmaceutical Preparation and Delivery, Institute of Process Engineering, Chinese Academy of Sciences, Beijing, China

*Corresponding author

Address correspondence to Y.Y at: yyuan@ipe.ac.cn, H.B. Shen at: [hbshen@sjtu.edu.cn](mailto:hbshen@sjtu.edu.cn)

**Supplementary Notes 1** | **Datasets**

The seqFISH dataset were processed according to the procedure reported in the original study. The resulting dataset consists of 125 genes measured in 1,597 cells. This dataset originates from mouse visual cortex. It contains a large number of neurons, glial cells, and endothelial cells.

For the MERFISH dataset, we used the Bregma+0.11 mm section area of animal No.18, which is the same with DeepLinc. After removed cells of unknown cell types, resulting in a final dataset of 160 genes measured across 4,975 cells. This dataset originates from mouse hypothalamic preoptic region. The dataset includes two types of neuronal cells—excitatory neurons and inhibitory neurons—as well as glial cells (Astrocytes, Endothelial 1/2/3, Oligodendrocytes Immature/Mature), immune cells (Microglia), and vascular-related cells (Ependymal and Pericytes).

The two HDST datasets used in this study are the mouse olfactory bulb (HDST_ob) and human breast cancer (HDST_cancer) tissue datasets, which is the same with DeepLinc. The olfactory bulb dataset contains 1,981 nuclei and 14,909 genes in total. The breast cancer dataset contains 1,765 nuclei and 8,336 genes. HDST_ob contains various cell types, including neurons, glial cells (Astrocytes, Ependymal, Oligodendrocytes, Peripheral Glia), immune cells (Immune), and vascular-related cells (Vascular). HDST_cancer includes a large number of immune cells (B cells, T cells, Macrophages), vascular cells (Endothelial), epithelial cells (Epithelial), and stromal cells (Stroma).

The last dataset is mouse olfactory bulb data obtained via the Stereo-seq technique. The Stereo-seq data were mapped to the cell-level resolution based on nucleus location, resulting in a dataset of 14,376 genes measured across 19,109 cells. This dataset contains Granule Cells (GC), Mitral/Tufted Cells (M/TC), Olfactory Sensory Neurons (OSN), Periglomerular Cells (PGC).

**Supplementary Notes 2** | **Definition of directed graphs.**

A directed graph can be described as a weighted dependency graph, *G*(*V*, *E*, w). *V* is the set of graph nodes. $E=\left\{ \left( i,j \right)\in V\times V:i\to j \right\}$ is the set of edges of the directed graph, denoted by pairs of nodes. $w:V\times V\mathbb{\to\mathbb{R}}$ is the weights of the edges, where $w\left( i,j \right)$ denotes the strength of the dependency relationship between *i*→*j*, and if $\left( i,j \right)\in E$, then $w\left( i,j \right)$ is zero. The adjacency of $G\left( V;E;w \right)$ can be represented as an $n\times n$ adjacency matrix with weights ***A***. This adjacency matrix has *m* nonzero entries and its $\left( i,j \right)$ entries are equal to their respective weights $w\left( i,j \right)$ i.e., $\boldsymbol{A}\left( i,j \right)=w\left( i,j \right)$. In the rest of this study, ${\tilde{\mathcal{N}}}^{+}\left( i \right)=\mathcal{N}^{+}(i)\bigcup\left\{ i \right\}$ and ${\tilde{\mathcal{N}}}^{-}\left( i \right)=\mathcal{N}^{-}(i)\bigcup\left\{ i \right\}$ are used to represent the set of outgoing and incoming neighboring nodes, ${deg}^{+}\left( i \right)=\left| {\tilde{\mathcal{N}}}^{+}(i) \right|$ and ${deg}^{-}\left( i \right)=\left| {\tilde{\mathcal{N}}}^{-}(i) \right|$ represent the outgoing and incoming degrees for node *i* including itself. Similar to the diagonal degree matrix $\tilde{\boldsymbol{D}}$ in an undirected, unweighted graph, the degree matrices corresponding to out-degrees and in-degrees on the diagonal of a directed graph are denoted as ${\tilde{\boldsymbol{D}}}^{+}$ and ${\tilde{\boldsymbol{D}}}^{-}$, representing the adjacency matrices with self-loops.

**Supplementary Notes 3** | **Identifying signature genes participated in short-range cell interactions.**

For each of the two datasets, we searched the literature on the top five genes with the highest sensitivity scores. Four of the top five genes in the human breast cancer dataset of HDST were found to be associated with cell-cell interactions, including IGHG1, KCTD2, RPS12, and SUCLA2. It was shown that IGHG1 is associated with cell adhesion, cytokine interactions and chemokine signaling pathways (Guohui, et al., 2019). Signaling through GPCRs is one of the most prominent mechanisms of cellular communication, which relies on signal transduction mediated by the binding of Gα and Gβγ to effector proteins. The homologous subfamilies of KCTD9 (KCTD2, KCTD5, and KCTD17) showed the strongest interactions with Gα and Gβγ in biological experiments, and this KCTD-Gβγ interaction has been used to probe the relationship between GPCR signaling in living cells and primary GPCR signaling in neurons (Sloan, et al., 2023). Proteins responsible for cell-cell adhesion are also enriched for succinyl lysine labeling, and in the absence of SCL, the driver of succinylation is the accumulation of succinyl-CoA in mitochondria due to mutations in SUCLA2 (Gut, et al., 2020).

In the mouse olfactory bulb dataset from HDST, four out of the top five genes with the highest sensitivity scores were found to be associated with cell adhesion and signaling, including COX7C, HSP90B1, Rhob, and ANKRD6. Rhob has been shown to be associated with cell growth control, actin regulation, adhesion-dependent viability, and gene expression (Lebowitz and Prendergast, 1998). miR-155, together with the target gene ANKRD6, is involved in adhesion junctions in cancer cells (Liu, et al., 2015). COX7C is a mitochondrial longevity protein that functions as a stable contact point between complexes I and IV essential for the assembly of complex IV and supercomplexes (Krishna, et al., 2021). HSP90B1 is a heat shock protein gene that is responsive to thyroid hormone and is involved in photoperiod signaling (Graham, et al., 2009).

**Supplementary Notes 4** | **Biological interpretation of learned representations**

To provide a more intuitive biological interpretation of the learned embeddings, we conducted a two-step significance analysis. First, we employed a gradient-based saliency-style method (Simonyan, et al., 2013) to estimate gene importance for S and T embeddings. This was achieved by calculating the gradient × input scores for each gene and averaging them across all cells. The top 40 most influential genes were identified for both S and T. Second, to determine whether these top-ranked genes were significantly enriched with ligands or receptors, a permutation-based enrichment test was performed. The observed proportions of ligand and receptor genes were compared against a null distribution generated by 10,000 random permutations of the gene set. We applied this analysis on HDST_ob, HDST_cancer and MERFISH datasets, as the seqFISH dataset contains only one overlapping L-R pair with the database. Our results consistently demonstrate that ligands are significantly enriched in the sender matrix S across all datasets, with empirical p-values of 0.001 (HDST_ob), 0.0289 (HDST_cancer), and 0.0336 (MERFISH), thereby validating the biological relevance of these embeddings. In contrast, the receiver matrix T shows no significant enrichment of receptor genes. This lack of enrichment likely stems from the fact that receptors are frequently regulated post-translationally and expressed at low, stable mRNA levels, limiting their detectability in transcriptomic data (Armingol, et al., 2021; Purvis and Lahav, 2013). Consequently, matrix T may represent a broader "receptive competence" through downstream signaling transducers rather than isolated receptor expression.

**Supplementary Notes 5** | **Cell annotation using learned representations**

To further evaluate the utility of learned representations in downstream cell type annotation, we utilized the Stereo-seq dataset (19,109 cells x 14,376 genes), the largest in our study. By comparing various feature combinations against raw gene expression across multiple classifiers (Random Forest, Support Vector Machine, and Logistic Regression), we observed that the concatenated representations consistently yielded the highest annotation accuracy (**Table S6**). Unsupervised clustering using K-means was also performed; the resulting Adjusted Rand Index (ARI) scores further confirm that our model's features capture cell identity more effectively than raw gene expression. UMAP visualizations (**Fig. S4**) further confirm that our model effectively filters technical noise and emphasizes biologically meaningful signals, producing more distinct and compact cell clusters compared to raw expression data.

**Supplementary Notes 6 | Memory Efficiency and Scalability**

To handle the massive scale of modern spatial transcriptomics, DualCellChat implements traditional memory-saving technique with sparse matrix representation. While dense adjacency matrices require O(N^2^) space, sparse tensors (e.g., CSR formats) are adopted instead. This reduces the memory footprint to O(E), where E is the number of edges (cell-cell interactions). E is typically linear relative to the number of cells N in spatial graphs.

To quantitatively assess how memory usage scales with cell numbers, we performed a profiling experiment using Stereo-seq data (19,109 cells x 14,376 genes). We sampled subsets of nodes (1000, 2000, 3000...) and recorded the peak memory consumption (**Fig. S5**). We then applied two fitting models to estimate the memory required for 1 million cells: the projected memory requirement ranges from a highly optimized 1.52 GB (log-linear fit) to a conservative maximum of 29.35 GB (quadratic fit). These memory requirements are well within an acceptable range for modern computing infrastructure.

**Table S1 Five spatial transcriptome datasets for DualCellChat testing.**

| Dataset | Cell number | Gene number | Tissue | Cell types |
| --- | --- | --- | --- | --- |
| seqFISH | 1,597 | 125 | mouse visual cortex | Endothelial  Glial Cells  Neuron |
| MERFISH | 4,975 | 160 | mouse hypothalamic preoptic region | Astrocyte Endothelial 1 Endothelial 2 Endothelial 3 Ependymal  Excitatory Inhibitory  Microglia  OD Immature 1  OD Immature 2  OD Mature 1  OD Mature 2  OD Mature 3  OD Mature 4  Pericytes |
| HDST_ob | 1,981 | 14,909 | mouse olfactory bulb | Astrocytes Ependymal  Immune  Neurons  Oligos PeripheralGlia  Vascular ambiguous |
| HDST_cancer | 1,765 | 8,336 | human breast cancer | Bcell  Tcell  endothelial epithelial macrophage  stroma  ambiguous |
| Stereo-seq | 14,376 | 19,109 | mouse olfactory bulb | Granule Cells  Mitral/Tufted Cells  Olfactory Sensory Neurons  Periglomerular Cells |

**Table S2 p-value results for DualCellChat performance stability under different noise intensities.**

| **Parameter σ** | **HSDT_ob p_value** | **HSDT_cancer_p_value** | **seqFISH p_value** | **MERFISH p_value** |
| --- | --- | --- | --- | --- |
| 1.0 | 0.682419542 | 0.988960315 | 0.070691626 | 0.751686324 |
| 1.1 | 0.520814988 | 0.862671555 | 0.074743283 | 0.558856184 |
| 1.2 | 0.446226434 | 0.861442376 | 0.016769587 | 0.891067858 |
| 1.3 | 0.444662331 | 0.851610594 | 0.009749726 | 0.249915698 |
| 1.4 | 0.499018814 | 0.936614771 | 0.000986536 | 0.211376494 |
| 1.5 | 0.425560175 | 0.538628161 | 3.54225E-05 | 0.048740583 |
| 1.6 | 0.425467854 | 0.388851234 | 1.82144E-05 | 0.000253641 |
| 1.7 | 0.48886366 | 0.324455136 | 1.02393E-05 | 8.28348E-05 |
| 1.8 | 0.648243146 | 0.429051063 | 8.13792E-06 | 9.67609E-06 |
| 1.9 | 0.802210001 | 0.484887227 | 0.003426428 | 1.38383E-06 |
| 2.0 | 0.660446142 | 0.295197505 | 0.002712539 | 5.64188E-07 |
| 2.1 | 0.880681393 | 0.360356089 | 0.000475664 | 7.91208E-08 |
| 2.2 | 0.491764704 | 0.55158325 | 6.58505E-07 | 1.48452E-08 |
| 2.3 | 0.394442885 | 0.32738464 | 2.35937E-05 | 3.31588E-08 |
| 2.4 | 0.184213002 | 0.175716679 | 0.000154268 | 3.88887E-08 |
| 2.5 | 0.148740609 | 0.188579603 | 0.001838896 | 4.17661E-09 |
| 2.6 | 0.074843019 | 0.106941795 | 3.78547E-06 | 5.94105E-08 |
| 2.7 | 0.029787552 | 0.072137005 | 1.19102E-06 | 5.7028E-08 |
| 2.8 | 0.019656861 | 0.111833523 | 1.61488E-07 | 6.65065E-10 |
| 2.9 | 0.00333911 | 0.163010719 | 1.22548E-06 | 5.66269E-08 |
| 3.0 | 0.000920018 | 0.256466324 | 1.4622E-07 | 1.04696E-09 |
| 3.1 | 0.001437702 | 0.285644497 | 1.8197E-06 | 1.2871E-10 |
| 3.2 | 0.001596338 | 0.097478973 | 2.79896E-06 | 7.20502E-10 |
| 3.3 | 0.000465371 | 0.034626505 | 6.54118E-06 | 1.47228E-09 |
| 3.4 | 0.000335657 | 0.004718836 | 3.02114E-07 | 9.17772E-11 |
| 3.5 | 0.000524723 | 0.003133482 | 9.12688E-07 | 1.02604E-06 |
| 3.6 | 9.77723E-05 | 0.003546951 | 2.05259E-08 | 6.88826E-10 |
| 3.7 | 0.000333622 | 0.00078174 | 3.73098E-09 | 8.76247E-06 |
| 3.8 | 1.30533E-05 | 0.000303454 | 4.67145E-09 | 2.18887E-06 |
| 3.9 | 2.73241E-05 | 5.30811E-05 | 8.47768E-08 | 4.71435E-08 |
| 4.0 | 3.20569E-06 | 1.52511E-07 | 1.73724E-12 | 4.2002E-07 |

**Table S3 Quantitative analysis of cell-type heterogeneity across data folds based on UMAP spatial dispersion.**

| **Fold** | **Total N Cells** | **Mean distance to center** | **Std distance to center** | **Mean UMAP Variance** | **Std UMAP Variance** | $\boldsymbol{\Delta}\mathbf{AUC}$ |
| --- | --- | --- | --- | --- | --- | --- |
| 0 | 10,113 | 1.534 | 0.989 | 2.911 | 1.637 | +0.017 |
| 1 | 10,149 | 1.478 | 0.953 | 2.533 | 1.234 | -0.008 |
| 2 | 10,162 | 1.498 | 1.022 | 2.810 | 1.450 | +0.011 |
| 3 | 10,080 | 1.498 | 0.977 | 2.573 | 1.333 | -0.006 |
| 4 | 10,211 | **1.535** | **1.013** | **3.034** | **1.797** | **+0.044** |

**Note:** $\Delta AUC=\mathrm{AUC}_{\mathrm{hetereogeneous}}-\mathrm{AUC}_{\mathrm{homogeneous}}$, representing the performance gain of the heterogeneous graph model over the homogeneous baseline. Std refers to standard deviation.

**Table S4 Cross-database validation results of DualCellChat predictions using CITEdb, CCIDB, and CCCdb databases.** This data is provided as a separate Excel file (Table S4.xlsx)

**Table S5 Identification and sensitivity ranking of ligand-receptor pairs across four spatial transcriptomics datasets.** This data is provided as a separate Excel file (Table S5.xlsx)

**Table S6 Performance comparison of cell type annotation using gene expression versus learned representations from DualCellChat.**

| **Features** | **Random Forest** | **SVM** | **Logistic Regression** | **ARI** |
| --- | --- | --- | --- | --- |
| Gene expression | 0.647 | 0.658 | 0.594 | 0.193 |
| S | 0.814 | 0.771 | 0.737 | 0.333 |
| T | 0.804 | 0.765 | 0.728 | 0.367 |
| Combined (S + T)/2 | 0.813 | 0.777 | 0.737 | 0.476 |
| Concatenated (S, T) | 0.816 | 0.777 | 0.750 | 0.480 |


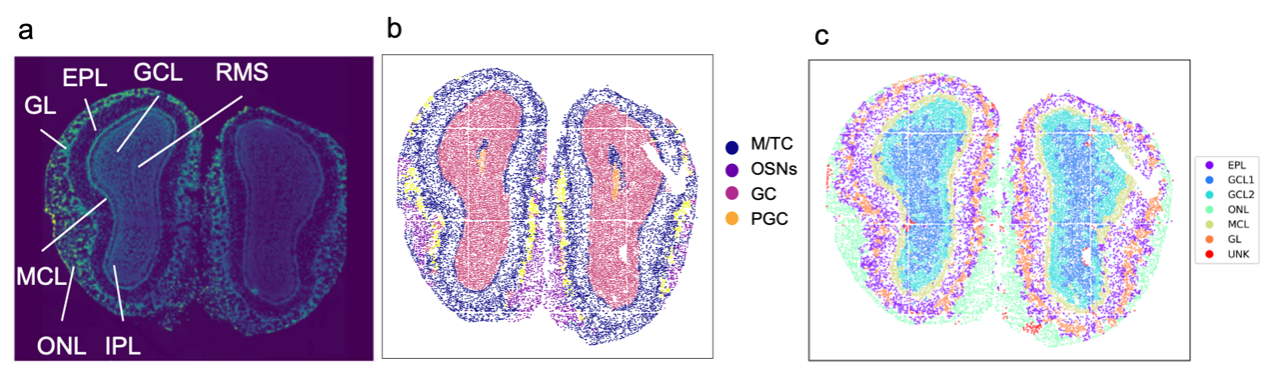


**Fig. S1 | Visualization of cell type annotation results. a.** The layered structure of the mouse olfactory bulb annotated in the DAPI-stained image produced by Stereo-seq. **b**. Visualization of cell type annotations generated by IRIS. **c**. Visualization of cell annotations generated by STAGATE.


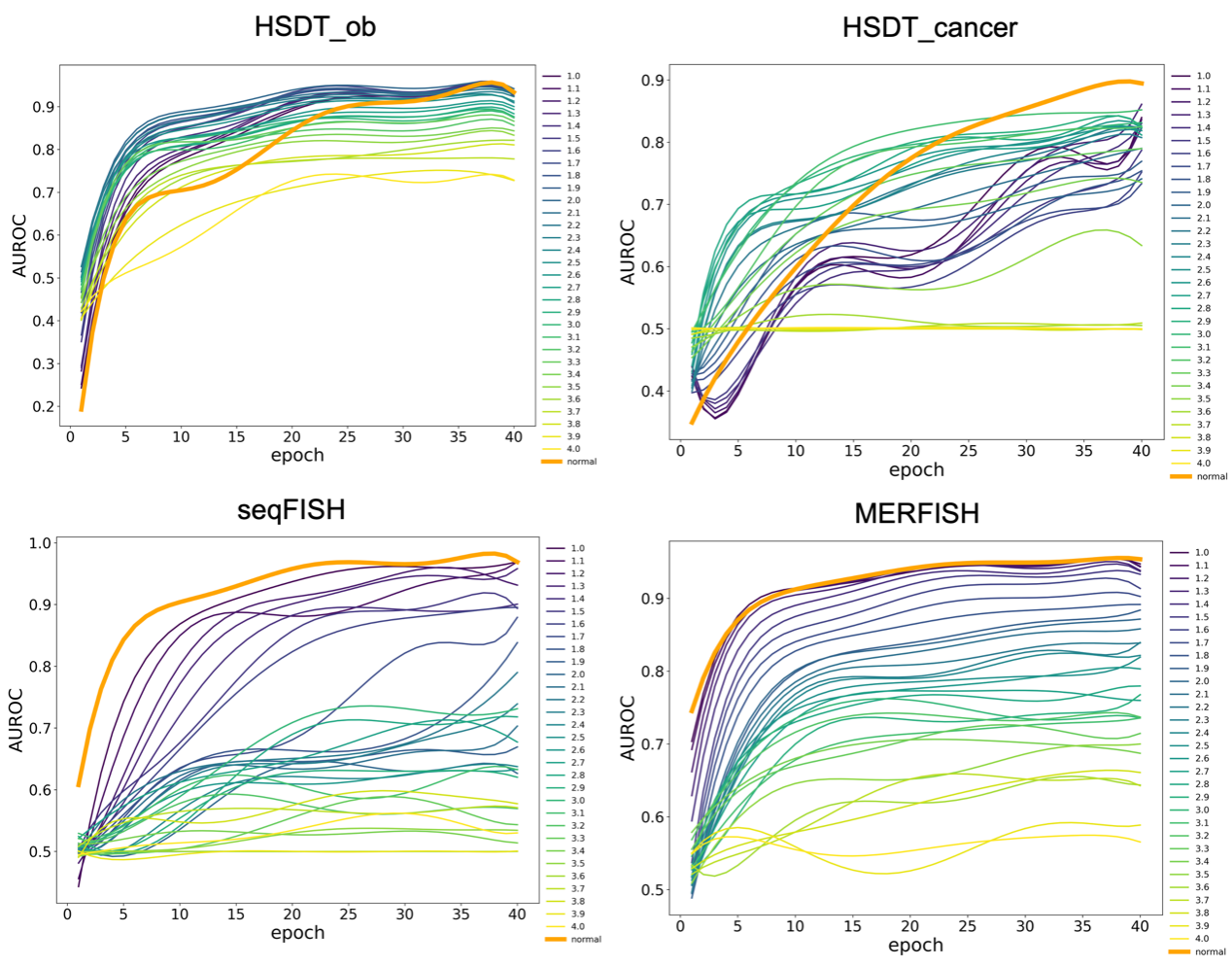


**Fig. S2 | Effect of artificial random noise with parameter σ, ranging from 1 to 4, on DualCellChat reconstruction results.**


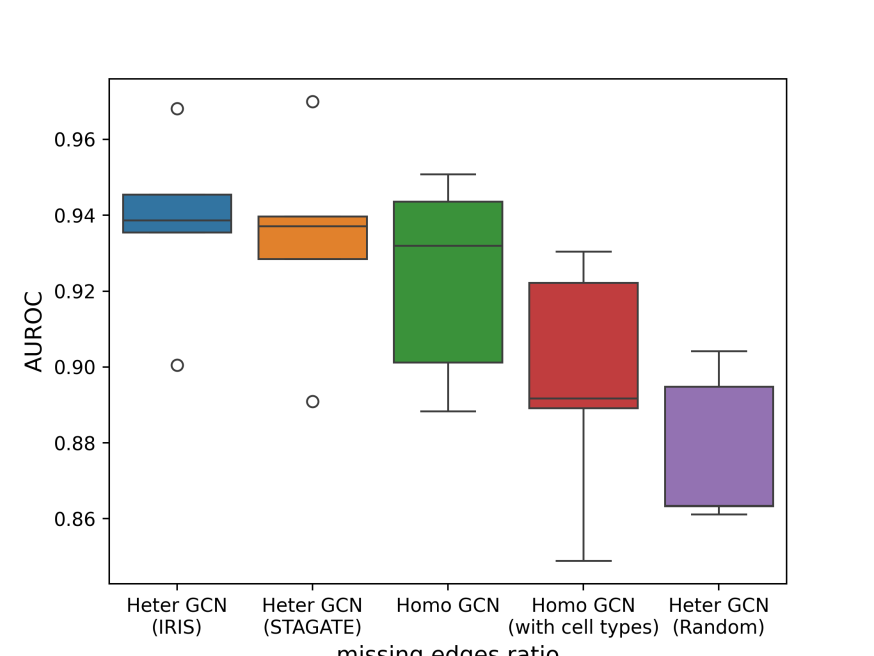


**Fig. S3 | Reconstruction results of incorporating cell type information using directed heterogeneous GCN on Stereo-seq data.** "Heter GCN" refers to Heterogeneous GCN, while "Homo GCN" stands for Homogeneous GCN. **The Heter GCN (IRIS)** uses cell type annotations from IRIS on a heterogeneous graph**. The Heter GCN (STAGATE)** uses cell annotations from STAGATE on a heterogeneous graph. **The Heter GCN (Random)** assigns cell types randomly on a heterogeneous graph. **Homo GCN** ignores cell types, **Homo GCN (with cell types)** incorporates cell types as node features.


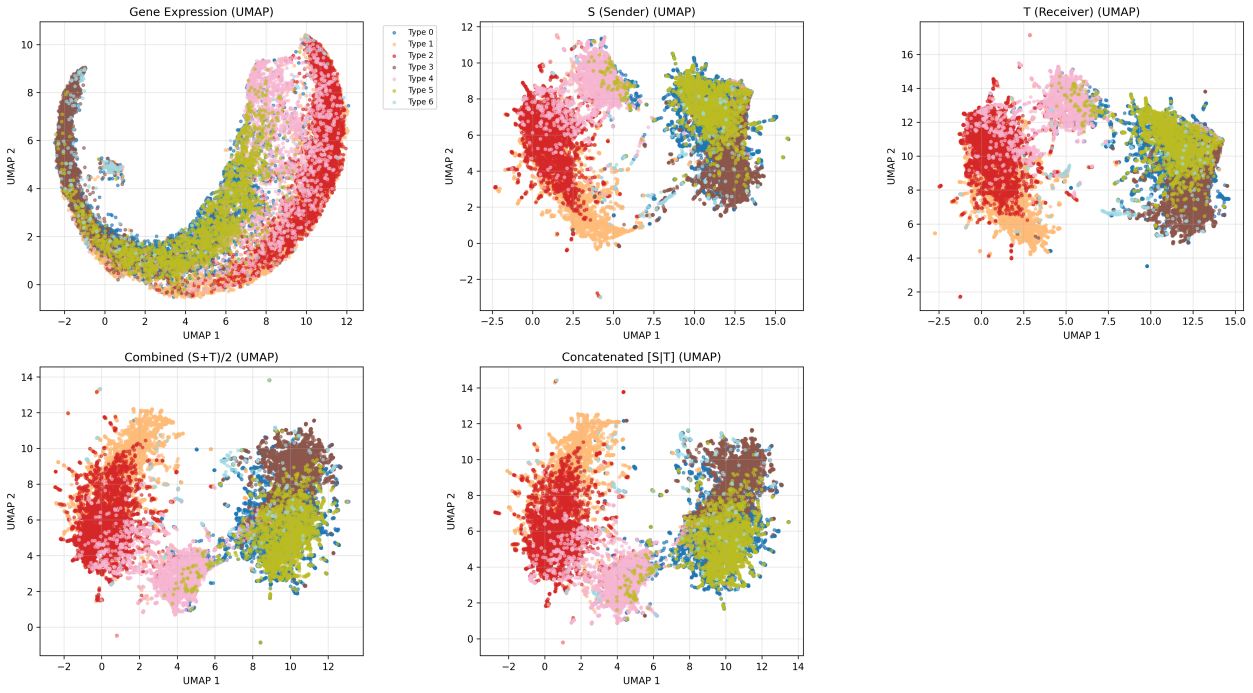


**Fig. S4 | UMAP Visualizations of Gene Expression, S, T, Combined and Concatenated features of S and T.**


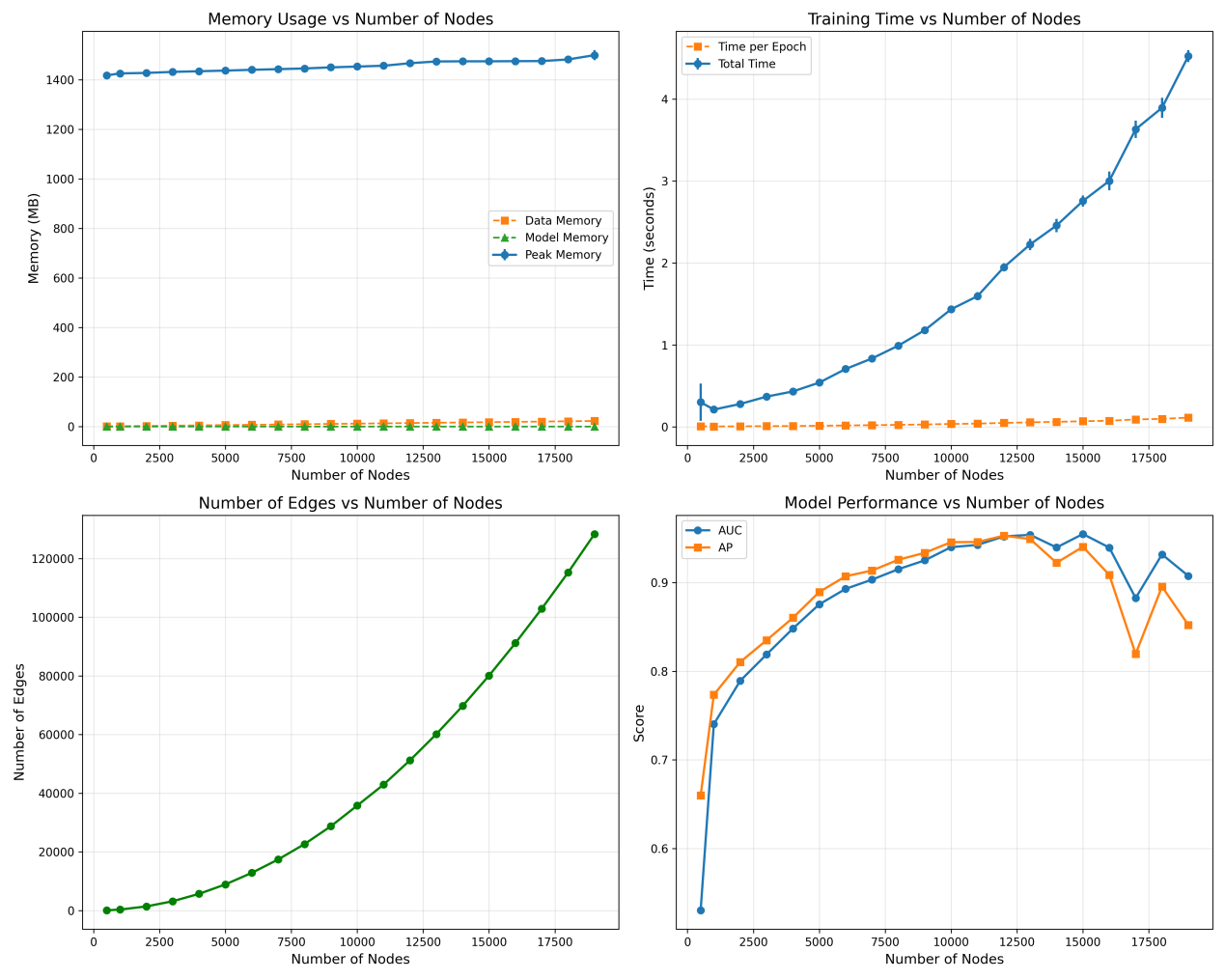


**Fig. S5 | Scalability analysis of peak memory usage across increasing cell counts.**

**References**

Armingol, E.*, et al.* Deciphering cell–cell interactions and communication from gene expression. *Nature Reviews Genetics* 2021;22(2):71-88.

Graham, G.*, et al.* HSP90B1, a thyroid hormone-responsive heat shock protein gene involved in photoperiodic signaling. *Brain research bulletin* 2009;79(3-4):201-207.

Guohui, W., Xiongtao, Y. and Peking, G.Z. Expression and Clinical Significance of Cancer-derived Immunoglobulin G in Non-small Cell Lung Cancer by Bioinformatics and Immunohistochemistry. *Chinese Journal of Lung Cancer* 2019;22(6).

Gut, P.*, et al.* SUCLA2 mutations cause global protein succinylation contributing to the pathomechanism of a hereditary mitochondrial disease. *Nature communications* 2020;11(1):5927.

Krishna, S.*, et al.* Identification of long-lived proteins in the mitochondria reveals increased stability of the electron transport chain. *Developmental Cell* 2021;56(21):2952-2965. e2959.

Lebowitz, P.F. and Prendergast, G.C. Functional interaction between RhoB and the transcription factor DB1. *Cell adhesion and communication* 1998;6(4):277-287.

Liu, J.*, et al.* Expression and function of miR-155 in breast cancer. *Biotechnology & biotechnological equipment* 2015;29(5):840-843.

Purvis, J.E. and Lahav, G. Encoding and decoding cellular information through signaling dynamics. *Cell* 2013;152(5):945-956.

Simonyan, K., Vedaldi, A. and Zisserman, A. Deep inside convolutional networks: Visualising image classification models and saliency maps. *arXiv preprint arXiv:1312.6034* 2013.

Sloan, D.C., Cryan, C.E. and Muntean, B.S. Multiple potassium channel tetramerization domain (KCTD) family members interact with Gβγ, with effects on cAMP signaling. *Journal of Biological Chemistry* 2023;299(3).
